# Quality of Life and Its Influencing Factors of Couples Referred to An Infertility Center in Shiraz, Iran

**DOI:** 10.22074/ijfs.2018.5123

**Published:** 2017-10-14

**Authors:** Bahia Namavar Jahromi, Mahsa Mansouri, Sedighe Forouhari, Tahere Poordast, Alireza Salehi

**Affiliations:** 1 Infertility Research Center, Shiraz University of Medical Sciences, Shiraz, Iran; 2 Department of Obstetrics and Gynecology, Shiraz School of Medicine, Shiraz University of Medical Sciences, Shiraz, Iran; 3 Student Research Center, Infertility Research Center, Shiraz University of Medical Sciences, Shiraz, Iran; 4 Shahid Sadoughi University of Medical Sciences, Infertility Research Center, Shiraz University of Medical Sciences, Shiraz, Iran; 5 MPH Department, Shiraz Medical School, Shiraz University of Medical Sciences, Shiraz, Iran

**Keywords:** Education, Income, Infertility, Quality of Life, Treatment

## Abstract

**Background:**

Infertility adversely affects quality of life (QoL). The present study aims to evaluate QoL and its associated factors among infertile couples.

**Materials and Methods:**

In this cross-sectional study, the Fertility QoL (FertiQoL) instrument was used to measure QoL among 501 volunteer couples who attended the Infertility Clinic at the Mother and Child Hospital, Shiraz, Iran. We used an additional questionnaire to assess participants’ demographic and clinical characteristics. The relationship between the scores of QoL to the sociodemographic and treatment data was analysed.

**Results:**

The subjects with lower income levels had lower relational, mind/body, emotional, and total core scores. Female participants without academic education had lower scores in the emotional subscale, while the male participants showed lower scores in emotional, mind/body, relational, social, and total QoL domains. Subjects who had undergone any type of treatment, including pharmacological treatment, intrauterine insemination (IUI), intra-cytoplasmic sperm injection (ICSI), and *in vitro* fertilization (IVF) showed significantly lower scores in the environmental domain. Participants with lower infertility duration obtained significantly greater QoL scores. Finally, tolerability, emotional, and environmental domains were significantly more desirable when the infertility problem was related to a male factor.

**Conclusion:**

Infertile couples with shorter duration of infertility and male etiology have higher QoL. Lower academic education, lower income levels, or prior unsuccessful treatments are associated with lower QoL.

## Introduction

Child bearing, one of the naturally desired human goals, is mandatory for survival of the human species. An estimated 72.4 million couples worldwide experience primary or secondary infertility (1). The reported infertility rate for different countries ranges from 5-30% (2). A meta-analysis has calculated an average infertility rate in Iran of 10.9% of the population (3). Infertility induces numerous psychological, economic, ethical, and cultural consequences that result in diminished self-confidence and quality of life (QoL) (4). Infertility and its related diagnostic or therapeutic modalities that include pharmacological treatment (oral pareneral administration), intrauterine insemination (IUI), *in vitro* fertilization (IVF), and intra-cytoplasmic sperm injection (ICSI) induce a heavy burden on affected couples. According to the World Health Organization (WHO) guidelines, ‘‘QoL is an individuals’ perception of their position in life in the context of culture and value systems in which they live’’ (5). A large number of studies have investigated QoL in infertile couples with non-specific questionnaires, such as World Health Organization Quality of Life form (WHOQOLBREF) and Health Survey Short Form (SF-36) (6,7). 

A few studies used the Fertility QoL (FertiQol) questionnaire as a fertility-specific QoL assessment of infertile individuals. The FertiQol questionnaire has been shown to be a valid, reliable measure for the impact of infertility on QoL (8,10). According to a substantial body of literature, infertility negatively affects QoL and appears to lead to mental problems, such as anxiety, depression, frustration, isolation, disturbed identity, and lack of attraction (11,13). Infertility is a critical issue for extended Iranian families (14). A study in Tehran has investigated QOL of infertile couples and reported significantly higher QoL in infertile men compared to infertile women (15). Until now, few studies have investigated factors that impact QoL in infertile couples. Of particular importance is how the factors that predict QoL vary in different infertile populations. Recognition of these associated factors is essential for program planning to increase the QoL of the infertile population (16,17). The present study aims to assess the QoL of infertile couples with particular emphasis on the influences of related factors on their QoL. 

## Materials and Methods

This cross-sectional study selected subjects by simple random sampling from infertile couples who attended the Infertility Clinic of the Mother and Child Hospital, Shiraz, Iran from February 2014 to March 2015. Couples who did not achieve pregnancy after at least one year of timed unprotected sexual intercourse were invited to voluntarily participate in this study. Overall, 501 eligible couples agreed to participate. All procedures were performed in accordance with the ethical standards of the Institutional and/or National Research Committee and with the 1964 Declaration of Helsinki, its later amendments or comparable ethical standards. 

The Ethics Committee of Shiraz University of Medical Sciences approved this study (code: 92-01-50-7132). At first, participants received a complete explanation about the nature of the study and each participant signed a written informed consent. All participants were requested to complete a data gathering form that contained structured questions about demographic information, socioeconomic status, and fertility characteristics. Interviews were also performed by the researchers and trained assistants for further explanations if needed and to assist the illiterate participants for their verbal consent and appropriate completion of the questionnaire in the Infertility Clinic. We administered the international FertiQol questionnaire to assess participants’ QoL (9). The questionnaires gathered information about age, educational and economic status, type of medications, causes of infertility, and duration of infertility. All parameters were considered to be the independent variable, whereas QoL was the dependent variable of the study. In this study, we used the Persian version of the FertiQoL instrument. The Persian version was previously proven to be a valid, reliable tool to evaluate QoL of infertile couples (10). 

The FertiQoL questionnaire consists of core and treatment sections: 24 specific questions that cover mind/ body, relational, social, and emotional domains in the core section and 10 questions on environment and tolerability domains in the treatment section. We divided the participants into three socioeconomic groups: low income (couples that made less than one million tomans per month); middle income (1-3 million tomans per month); and high income (more than 3 million tomans per month). Scores of the six subscales of FertiQoL instrument could range from 0 to 100, where higher scores indicated a better QoL. 

### Statistical analysis

The scores of QoL were calculated by the Researchers Excel scoring FertiQol online system. Statistical analyses were performed using SPSS 18.0 statistical software (SPSS, Inc., Chicago, IL, USA). Continuous variables were compared using the independent sample t test, Mann-Whitney U test, and one-way ANOVA. Additionally, categorical variables were compared using Pearson’s chi-square test. The relationship between the scores of every scale and subscale of QoL with the sociodemographic and treatment data was analyzed. P<0.05 were considered significant. 

## Results

Out of the 501 eligible infertile couples, 499 couples properly completed the questionnaires. The mean age of the male participants was 32.30 ± 5.65 years and for female participants, it was 31.66 ± 6.13 years. The self-reported monthly incomes of 389 (78.1%) couples were equal or less than one million tomans, 87 (17.5%) couples earned 1-3 million tomans, and 11 (2.2%) couples earned more than 3 million tomans per month. In this survey, 11 cases did not report their monthly incomes. The participants’ educational and fertility characteristics are listed in Table 1. 

**Table 1:** Educational and fertility characteristics of the participants


Variable		n (%)

Cause of infertility		
	Male	147 (29.5)
	Female	132 (26.5)
	Both	45 (9.0)
	Unexplained	130 (26.1)
Duration of infertility		
	<5 Y	302 (60.6)
	5-10 Y	101 (20.3)
	>10 Y	63 (12.7)
Education (male)		
	<9 Y	89 (18.5)
	9-11 Y	221 (46.0)
	>11 Y	170 (35.4)
Education (female)		
	<9 Y	67 (13.9)
	9-11 Y	217 (45.2)
	>11 Y	196 (40.8)


scores among the women with lower academic degrees (Table 2). The men with lower academic degrees also obtained significantly lower scores in the total FertiQol, mind/body, relational, social, and emotional domains. Total FertiQol, total treatment, tolerability, environmental, and emotional subscales had better scores when the male factor was the cause of infertility compared to the conditions where females or both sexes were the causes for infertility. 

Treatment of infertility, including pharmacological treatment (oral or parenteral medications), as well as IUI, IVF, and ICSI had lower scores for total treatment, tolerability, and environmental domains compared to the cases that did not use medications (P<0.05). 

## Discussion

In the present study, we have sought to evaluate the sociodemographic and clinical variables that influenced QoL in infertile couples. In the general Iranian population, a woman’s social standing is strongly tied to maternal status or the possibility of pregnancy. Motherhood experience increases the total QoL score. FertiQoL is a reliable, sensitive instrument to evaluate QoL in infertile couples. In a recently published study, Maroufizadeh et al. (10) have evaluated the validity and reliability of the Persian version of the FertiQol instrument. They concluded that the Persian version performed similar to the original English version. They tested the factor structures of the FertiQol Instrument and suggested removal or modifications for Q15 and T2 to gain a higher loading on internal consistency of the relational and environmental factors. 

The FertiQol instrument covers QoL with respect to treatment quality and tolerability (9). Despite the advantages over other techniques, a few studies have used the FertiQoL instrument to assess QoL in terms of infertility (6,8). In a review, Mousavi et al. (7) assessed different general and specific questionnaires used to evaluate QoL in infertile people. They found that researchers used 10 general and 2 specific questionnaires to assess QoL of infertile patients in the literature. They concluded that the two most frequently used general questionnaires were SF-36 and WHO-QOL. FertiQoL and the Fertility Problem Inventory, as specific tools, were seldom used. 

Evaluation of QoL in both infertile partners was one of the strong points of this study. Our findings showed that total FertiQol, total treatment, tolerability, environmental, and emotional subscales had better scores when the male partners were the causes of infertility. Several investigators previously evaluated the role of male factor infertility on the QoL of couples (18,19) and found less negative effects of infertility on the men’s and couples lives as evidenced by significantly higher QoL compared to those with female factor infertility (20,22). Huppelschoten et al. (23) concluded that infertile women had lower fertility-related levels of QoL and were at increased risk for developing emotional problems compared to their partners. However, Chachamovich et al. (24) examined infertile couples’ QoL and found no differences between male and female partners. Rashidi et al. (15), in a cross-sectional study, assessed infertile couples’ QoL using the SF-36 and concluded that the causes of infertility did not have significant effects on health-related QoL in infertile couples. These differences might be related to the use of a fertility-specific instrument (FertiQoL) in the study by Huppelschoten et al. (23) and the current study compared to the generic QoL assessment instrument by Chachamovich et al. (24) and Rashidi et al. (15). Therefore, further research should be performed to confirm the impact of male or female infertility on couples’ QoL. 

**Table 2:** The effects of demographic and other variables on quality of life (QoL) in infertile couples


Study variable		Total Treatment score(mean ± SD)	Emotional subscale(mean ± SD)	Relational subscale(mean ± SD)	Mind-body subscale(mean ± SD)	Social subscale (mean ± SD)	Total Core score(mean ± SD)	Treatment Environment subscale(mean ± SD)	Treatment Tolerabilitysubscale(mean ± SD)	Total FertiQol score(mean ± SD)	

Treatment	Pharmacological	51.9 ± 22.2	58.7 ± 17.2	58.1 ± 24.1	61.6 ± 18.2	53.6 ± 13.7	55.5 ± 20.8	54.2 ± 14.7	60.1 ± 18.1	58.4 ± 15.6
	Surgery	50.2 ± 24	57.8 ± 14.6	57.6 ± 23.1	59.9 ± 19.3	53.3 ± 16.2	54.6 ± 20.8	53.8 ± 15.1	59.2 ± 18.3	57.3 ± 15.8
	IUI	45.7 ± 21.7	55.3 ± 13.4	52.8 ± 21.8	59.8 ± 17.0	53.7 ± 15.6	51.5 ± 22	53 ± 15.3	55.7 ± 15.8	54.8 ± 13.8
	IVF, ICSI	49.2 ± 18.8	57.9 ± 14.8	57.2 ± 21.6	61.3 ± 19.3	54.1 ± 17.8	51.5 ± 19.1	52.9 ± 15.6	59.1 ± 15.7	57.3 ± 13.0
	No medication	53.1 ± 23.1	58.7 ± 15.2	61.8 ± 26.6	59.3 ± 21.4	61.4 ± 14.5	62.1 ± 24.8	62 ± 15.2	61.3 ± 18.6	61.1 ± 16.2
	P value	0.183	0.224	0.179	0.945	0.021	0.036	0.005	0.220	0.159
Monthly income	Below 1 million T^*^	49.3 ± 22.1	56.3 ± 15.1	56.5 ± 23.4	59.7 ± 18.8	55.2 ± 16.3	55.8 ± 22	55.5 ± 15.7	57.9 ± 17.1	57.1 ± 15.1
	Above 1 million T^*^	58.4 ± 22.0	63.8 ± 14.7	67.5 ± 21.8	67.7 ± 15.8	57.8 ± 14.7	59 ± 22.3	58 ± 15.2	67.1 ± 15.8	64.6 ± 14.0
	P value	0.001	0.001	0.001	0.000	0.287	0.001	0.101	0.00	0.001
Duration of infertility	<5 Y	50.6 ± 21.9	58.8 ± 15.2	58.9 ± 23.2	62 ± 17.8	56.4 ± 15.1	57.6 ± 22.1	56.8 ± 14.8	60.2 ± 16.7	59.1 ± 14.7
	5-10 Y	48.5 ± 22.4	54.7 ± 15.6	53.4 ± 22.8	59 ± 19.5	54.9 ± 18.2	52.3 ± 21.2	54 ± 17.6	56.4 ± 18.0	55.7 ± 15.6
	≥10 Y	52.3 ± 23.7	55.9 ± 14.8	60.1 ± 24.4	58.8 ± 21.1	51.2 ± 16.2	52.8 ± 20.6	52 ± 14	59.2 ± 18.5	57.1 ± 15.3
	P value	0.549	0.073	0.092	0.257	0.064	0.050	0.048	0.166	0.107
Education	Men									
	<9 Y	45.5 ± 22.8	52.7 ± 14	55.2 ± 24	57.7 ± 19.3	57.6 ± 14.7	56.1 ± 23.5	57.1 ± 16.5	55.2 ± 17.3	55.4 ± 15.4
	9-11 Y	49.3 ± 21.5	56.9 ± 15.3	56.7 ± 23.7	60.2 ± 19.1	55.2 ± 15.9	56.7 ± 22.3	56.1 ± 15.1	58.3 ± 17.1	57.6 ± 15.1
	>11 Y	55.7 ± 22.6	61.2 ± 15.2	62.8 ± 22.2	64.2 ± 17.4	55.5 ± 16.9	56.9 ± 21.2	55.8 ± 16	63.7 ± 16.7	61.5 ± 14.5
	P value	0.001	0.000	0.014	0.015	0.471	0.907	0.848	0.00	0.003
	Women									
	<9 Y	46 ± 22.0	53.9 ± 13.3	55.5 ± 23.3	59.3 ± 19.2	57.3 ± 13.9	62.4 ± 23.1	59.6 ± 15.4	56.1 ± 16.3	56.8 ± 14.55
	9-11 Y	49.5 ± 21.6	56.1 ± 15.5	56.2 ± 24	59.3 ± 19.5	56.6 ± 16.2	55.8 ± 22.5	56.4 ± 15.7	57.8 ± 17.5	57.3 ± 15.2
	>11 Y	54.7 ± 22.8	61.2 ± 14.9	62.7 ± 22.3	64.5 ± 17.4	54.3 ± 16	55.9 ± 20.8	54.8 ± 15.2	63.6 ± 16.7	61.1 ± 14.8
	P value	0.012	0.00	0.008	0.010	0.237	0.072	0.093	0.00	0.015
Causes of infertility	Male factor	53.5 ± 21.2	58.4 ± 14.9	61.2 ± 22.7	61.6 ± 18.2	59.1 ± 14.5	59.8 ± 21.2	59.3 ± 14.1	61.4 ± 16.3	60.7 ± 14
	Female factor	47.7 ± 23.0	57.5 ± 15.9	55.4 ± 23.6	60.5 ± 19.6	54.1 ± 16.3	51.8 ± 21.1	53.1 ± 15.4	57.7 ± 18.0	56.4 ± 15.7
	Both	46 ± 19.9	56.3 ± 15.4	54.5 ± 21.5	58.6 ± 20.7	52.9 ± 14	53.4 ± 21.9	53.2 ± 15.3	56.4 ± 16.6	55.4 ± 14.6
	Unexplained	53.3 ± 22.7	58.7 ± 14.7	61.1 ± 24	63.5 ± 16.9	54.2 ± 16.4	57.4 ± 22	55.4 ± 15.4	61.8 ± 17.2	59.8 ± 15.3
	P value	0.034	0.793	0.067	0.351	0.016	0.010	0.005	0.085	0.036


IUI; Intrauterine insemination, IVF; In vitro fertilization, ICSI; Intra-cytoplasmic sperm injection, and *; Toman.

Karabulut et al. (25) reported that tertiary education was related to higher scores in the total, emotional, and environment domains of QoL. In other previous studies that used SF-36 for health-related QoL and WHOQOL-BREF for general QoL, the women with lower educational levels scored worse in the vitality, environment, and mental health domains (16). Similarly, our study showed significantly lower emotional scores in women with lower educational levels. The scores of the total FertiQol, mind/body, relational, social, and emotional domains were also significantly lower among the men with lower academic education. The current study results showed that couples with shorter duration of infertility had higher scores in the total treatment domain, which confirmed results from two previous studies (25,26). However Chachamovich et al. (16) used WHOQOL-BREF and discovered no difference among the groups with different infertility durations. According to Rashidi et al. (15), SF-36 questionnaire results indicated that duration of infertility had no significant effects on health-related QoL in infertile couples. These differences might be due to different instruments used for the surveys or differences in the study groups’ characteristics. The subject women in the study by Chachamovich et al. (16) had higher educational levels compared to the women that participated in the current study. 

Researchers have noted an association between low adherence to fertility treatments and psychological imbalances (27,28). Boivin et al. (29) discovered that women with a moderate number of treatments exhibited more stress compared to their counterparts that received no treatment or who underwent treatment for a significant amount of time. Our results showed lower QoL scores in the subjects who had undergone treatment compared to those who had not yet began treatment. Ragni et al. (26) noticed that IVF treatment was accompanied by lower scores in the mental health domain. In another study, the couples who had IVF treatments had greater emotional disturbances and anxiety compared to the control group (30). Similarly, Chachamovich et al. (16) revealed that awaiting IVF was associated with lower scores in the vitality and psychological domains. They observed that other treatments did not share a similar influence on the QoL domains. However, our study findings indicated that treatments such as IUI, IVF, and ICSI had comparable impacts on the QoL domains. 

Our findings indicated that QoL scores were higher in infertile couples with shorter durations of infertility and male factor infertility. Lower education status and income levels, in addition to prior unsuccessful treatments were associated with lower QoL scores. We believe that comprehensive evaluation of influencing factors on the QoL of infertile couples using a fertility-specific questionnaire might help policy makers to detect and appropriately plan for infertile couples to receive the necessary suitable economical, psychosocial, and medical supports and to increase the accessibility of the treatment modalities. We propose complete insurance coverage of infertility treatments, at least for couples who do not have any offspring, in order to decrease the economic burden. An expert psychological support team should be readily available in infertility clinics for the affected couples to increase their living-skills and sense of satisfaction with life. 

## Conclusion

Infertile couples with shorter duration of infertility and male etiology have higher QoL. Lower academic education, lower income levels, or prior unsuccessful treatments are associated with lower QoL. 
