# Quality of Life and Its Influencing Factors of Couples Referred to An Infertility Center in Shiraz, Iran

**DOI:** 10.22074/ijfs.2018.5460

**Published:** 2018-01-15

**Authors:** Bahia Namavar Jahromi, Mahsa Mansouri, Sedighe Forouhari, Tahere Poordast, Alireza Salehi

**Affiliations:** 1 Infertility Research Center, Shiraz University of Medical Sciences, Shiraz, Iran; 2 Department of Obstetrics and Gynecology, Shiraz School of Medicine, Shiraz University of Medical Sciences, Shiraz, Iran; 3 Student Research Center, Infertility Research Center, Shiraz University of Medical Sciences, Shiraz, Iran; 4 Shahid Sadoughi University of Medical Sciences, Infertility Research Center, Shiraz University of Medical Sciences, Shiraz, Iran; 5 MPH Department, Shiraz Medical School, Shiraz University of Medical Sciences, Shiraz, Iran

In this article which was published in Int J Fertil Steril, Vol 11, No 4, Jan-Mar 2018, on pages 293-297, the “Duration of tducation (male)” was misspelled in Table 1. The corrected one is “Duration of education (male)”.

**Table 1:** Educational and fertility characteristics of the participants


Variable		n (%)

Cause of infertility		
	Male	147 (29.5)
	Female	132 (26.5)
	Both	45 (9.0)
	Unexplained	130 (26.1)
Duration of infertility		
	<5 Y	302 (60.6)
	5-10 Y	101 (20.3)
	>10 Y	63 (12.7)
Duration of education (male)		
	<9 Y	89 (18.5)
	9-11 Y	221 (46.0)
	>11 Y	170 (35.4)
Duration of education (female)		
	<9 Y	67 (13.9)
	9-11 Y	217 (45.2)
	>11 Y	196 (40.8)


In the sentence “These differences might be related to the use of a fertility-specific instrument (FertiQoL) in the study by Huppelschoten et al. (23) and the current study compared to the general QoL assessment instrument by Chachamovich et al. (24) and Rashidi et al. (15).” Which was at the page of 296 in the discussion section, the word “generic” was corrected in to “general”.

